# Holmium laser enucleation of the prostate: surgical, functional, and quality-of-life outcomes upon extended follow-up

**DOI:** 10.1590/S1677-5538.IBJU.2014.0561

**Published:** 2016

**Authors:** Ilter Alkan, Hakan Ozveri, Yigit Akin, Tumay Ipekci, Yusuf Alican

**Affiliations:** 1 Department of Urology, Okmeydani Teaching and Research Hospital, Istanbul, Turkey; 2 Department of Urology, Acibadem University School of Medicine, Kozyatagi, Istanbul, Turkey; 3 Deparment of Urology, Harran University School of Medicine, Sanliurfa, Turkey; 4 Department of Urology, Baskent University School of Medicine, Alanya Teaching and Research Hospital, Alanya, Antalya, Turkey; 5 Department of Urology, Prosmed Clinic, Nisantasi, Istanbul, Turkey

**Keywords:** Prostatic Hyperplasia, Holmium, Laser Therapy, Prostate, Quality of Life

## Abstract

**Objectives:**

To evaluate the long-term surgical, functional, and quality-of-life (QoL) outcomes after Holmium laser enucleation of the prostate (HoLEP) in patients with symptomatic benign prostatic hyperplasia (BPH).

**Materials and Methods:**

We retrospectively reviewed recorded data on patients who underwent HoLEP between June 2002 and February 2005. Ninety-six patients were enrolled. Demographic, perioperative, and postoperative data were recorded. On follow-up, International Prostate Symptom Scores (IPSSs), prostate-specific antigen (PSA) levels, QoL scores, peak uroflowmetric data (Q_max_ values), and post-voiding residual urine volumes (PVR volumes), were recorded. Complications were scored using the Clavien system. Statistical significance was set at p<0.05.

**Results:**

The mean follow-up time was 41.8±34.6 months and the mean patient age 73.2±8.7 years. The mean prostate volume was 74.6±34.3mL. Significant improvements in Q_max_ values, QoL, and IPSSs and decreases in PSA levels and PVR volumes were noted during follow-up (all p values=0.001). The most common complication was a requirement for re-catheterisation because of urinary retention. Two patients had concomitant bladder tumours that did not invade the muscles. Eight patients (8.3%) required re-operations; three had residual adenoma, three urethral strictures, and two residual prostate tissue in the bladder. Stress incontinence occurred in one patient (1%). All complications were of Clavien Grade 3a. We noted no Clavien 3b, 4, or 5 complications during follow-up.

**Conclusions:**

HoLEP improved IPSSs, Q_max_ values, PVR volumes, and QoL and was associated with a low complication rate, during extended follow-up. Thus, HoLEP can be a viable option to transurethral resection of the prostate.

## INTRODUCTION

Benign prostatic hyperplasia (BPH) is common in elderly males (1). Medical treatment is usually the first choice (2). If this is inadequate, surgery may be required (3). Transurethral re-section of the prostate (TURP) has long been the accepted gold standard to treat symptomatic disease of prostates weighing 30-80g (3). Open prostatectomy may be used to treat larger prostates (3). However, TURP may cause TURP syndrome, and open surgery may trigger gross bleeding, delayed recovery, and a decrease in the quality-of–life (QoL). Efforts to develop minimally invasive surgeries are continuing. Laser therapy afforded a quantum jump in such efforts; laser-mediated prostate enucleation is now commonplace (3) and has recently been accepted as an alternative treatment for BPH (4). Specifically, holmium laser enucleation of the prostate (HoLEP) is used to treat enlarged prostates, particularly in patients taking anticoagulants (5). HoLEP is safe and the complication rate acceptable (6); HoLEP afforded very good results when used to treat symptomatic BPH (4–6). Many relevant publications have appeared (7). However, although several studies featured 3-10 years of follow-up, longer-term follow-up data are lacking.

Herein, we evaluated the long-term surgical and functional results obtained by HoLEP, in patients with symptomatic BPH. Additionally, complications and QoL were evaluated. To the best of our knowledge, this is first study to evaluate QoL as an outcome during extended follow-up.

## MATERIALS AND METHODS

We retrospectively evaluated prospective data recorded by two surgeons (I.A. and Y.A.). All patients gave written informed consent and the Institutional Review Board approved the study. A total of 114 patients underwent HoLEP between June 2002 and February 2005. Exclusion criteria were the absence of follow-up data, previous prostate surgery, prostate cancer (PCa), and/or a neurological disease. If a suspicious prostate nodule was evident upon digital rectal examination (DRE), and/or the prostate specific antigen (PSA) level was elevated (>4ng/mL), prostate biopsy guided by transrectal ultrasonography was performed. Patients with PCa, developed either before or after HoLEP, were excluded. All patients were continent prior to operation. Finally, 96 patients were enrolled.

### Patient Data

Demographic data included age (in years), previous operations, co-morbidities, and medications taken. Preoperative data included prostate volume; uroflowmetric parameters including the maximum flow rate (Q_max_); post-voiding residual urine volume (PVR volume; mL), score on the Turkish version of the International Prostate Symptom Score (IPSS) (8); serum PSA level (ng/dL), DRE findings, and QoL questionnaire responses. Perioperative data included operation time; enucleation time; morcellation time; and the weight of enucleated tissue. Postoperative data consisted of hospital stay (days); duration of urethral catheterisation; pathology findings; and complications evaluated using the Clavien-Dindo system (9). To assess QoL, we used the World Health Organization Quality of Life Scale (Brief Version). QoL questionnaires were completed both before operation and during the follow-up period.

#### The operation

Gilling et al. have described the HoLEP procedure (10). All operations were performed using a 100W Ho: Yag laser fitted with a 550μm end-firing fibre (Lumenis^®^, Santa Clara, CA, USA) and 25.6-or 27-Fr continuous-flow resectoscopes with modified working elements (25.6 Fr: ACMI^®^, Southborough, MA, USA; 27 Fr: Olympus, Hamburg, Germany). The laser fibre was stabilised within a 6-Fr ureteral catheter and inserted into the channel of the modified working element. A 26-Fr nephroscope (Storz^®^, Tuttlingen, Germany) was used for morcellation. All procedures were performed under regional anaesthesia.

**Figure-1:**
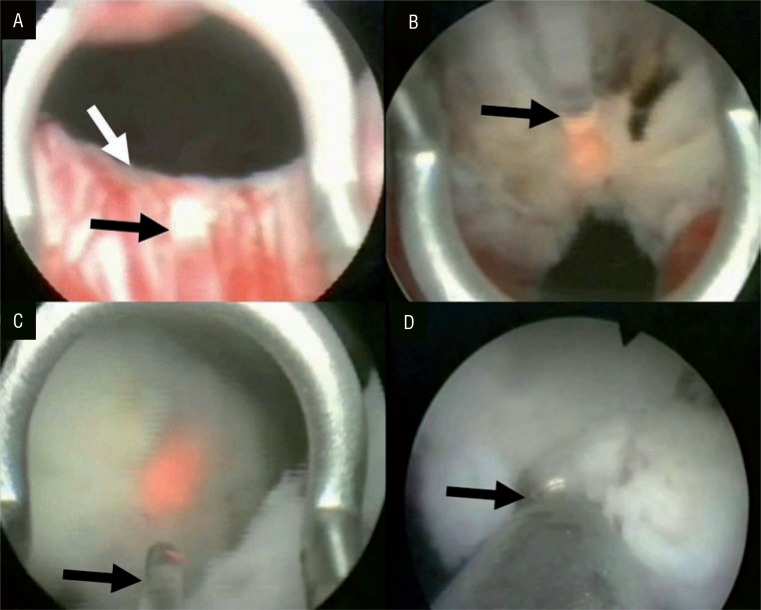
Enucleation and morcellation were performed respectively during operation. A) Incisions were performed from bladder neck to verumontanum level in terms of enucleating prostate lobes. Black arrow shows laser probe and white arrow shows bladder neck, B) An incision was performed from the bladder neck to the verumontanum level at 12 o'clock position, black arrow shows laser probe, C) Incisions were continued to the bladder neck and lateral lobe enucleation was completed; black arrow shows laser probe, D) Then, morcellation was performed; black arrow shows the tip of morcellator.

Briefly, the urethra was calibrated to 30Fr and the resectoscope inserted (after positioning) in the operating theatre. Room-temperature saline was used for irrigation. The preferred laser settings were 2J at 50Hz, or 2J at 40Hz. Initially, two incisions were performed at the 5 and 7 o'clock positions, lateral to the median lobe, from the neck of the bladder to the verumontanum, using the 550μm laser fibre. The distal ends of the incisions were joined just before the level of the verumontanum, and retrograde cutting continued to the neck of the bladder, to enucleate the median lobe (Figure- 1A). The incision was then extended upward, beneath the lateral lobe, to the 7 o'clock level of the verumontanum. Another incision was created from the neck of the bladder to the 12 o'clock position of the verumontanum, enucleating the left lateral lobe (Figure-1B). The incisions were joined just before the level of the verumontanum, and cutting continued retrograde to the bladder neck, to complete enucleation of the lateral lobe (Figure-1C). The same technique was used to enucleate the right lobe. Next, the prostatic fossa was checked for bleeding, and any necessary coagulation performed using a laser beam delivering 2J at 40Hz. A Versacut tissue morcellator (Lumenis^®^) was used to remove enucleated prostate tissue from the bladder. A specifically designed laser resectoscope fitted with a Morce-scope set (Wolf^®^, Knittingen, Germany) were used to treat the last 20 cases (this equipment became commercially available only recently). Large lobes were removed from the bladder after morcellation. It is essential to ensure good vision and adequate bladder distention during morcellation, to prevent bladder wall entrapment by the morcellator blades (Figure-1D). At the end of the procedure, a 20-Fr two-way Foley catheter was in-dwelled; this was removed 1 day later if haematuria was absent.

#### Follow-up

Follow-up was conducted at 1, 3, and 6 months after operation, and yearly thereafter. IPSSs, uroflowmetric data, serum PSA level, PVR volume, QoL, and IIEF data were evaluated.

Continence status was investigated using the short form of the International Continence Society. Incontinence was defined as a need for more than one protective pad daily (11). Complete dryness or the need for less than one pad daily was accepted as continence. If an erection adequate to engage in sexual intercourse was attained, with or without medication, the patient was considered potent. All patients completed International Index of Erectile Function (IIEF) questionnaires before and after Ho-LEP. If the IIEF-5 score of a patient was≤11, that patient was considered to exhibit erectile dysfunction. Continence and erectile status were assessed every year during follow-up. However, both surgeons changed their workplace, and the vast majority of patients were lost to follow-up at 6 and 7 years.

#### Statistical analyses

The Statistical Package for the Social Sciences (SPSS) for Windows, version 16.0 (SPSS Inc., Chicago, IL), was used for statistical analysis. The independent samples t-test was employed to compare continuous data, and one-way analysis of variance was also used for between-group comparisons. Statistical significance was set at p<0.05.

## RESULTS

The mean follow-up time was 41.8±34.6 months and the mean patient age 73.2±8.7 years. The mean prostate weight was 74.6±34.3g. Ninety patients (93.75%) were taking oral alpha-blockers to treat BPH. Additionally, 41 patients were taking the 5-alpha reductase inhibitor dutasteride. Demographic and preoperative data are summarised in Table-1.

**Table 1:** Demographic and perioperative data of study group.

Parameter	Mean±SD
Age (years)	73.2±8.7
PSA (ng/dL)	4.5±0.3
IPSS	22.1±6.1
Qmax (mL/sec.)	10.8±4.8
Prostate volume (g)	74.6±34.3
PVR	219.8±220.9
QoL score	4.6±1.2

**IPSS =** International prostate symptom score; **PSA =** Prostate specific antigen; **PVR =** Post voiding residual urine volume; **SD =** Standard deviation; **Q_max_ =** Maximum flow rate in uroflowmetry; **QoL =** Quality of life.

The mean operation time was 97±42.2 min including a mean morcellation time of 17.8±15.1 min. The mean weight of enucleated tissue was 29.2±11.3g. The mean duration of catheterisation was 1.2±0.8 days and the mean hospital stay 1.1±0.4 days (Table-2).

**Table 2:** peri- and post-operative data.

Parameter	Mean±SD
Operation time (min.)	97±42.2
Energy (kj)	206.8±131.96
Morcellation time (min.)	17.8±15.1
Volume of enucleated tissue (g)	29.2±11.3
Duration of catheter (day)	1.2±0.8
Hospital stay (day)	1.1±0.4

**SD =** Standard deviation.

**Figure-2:**
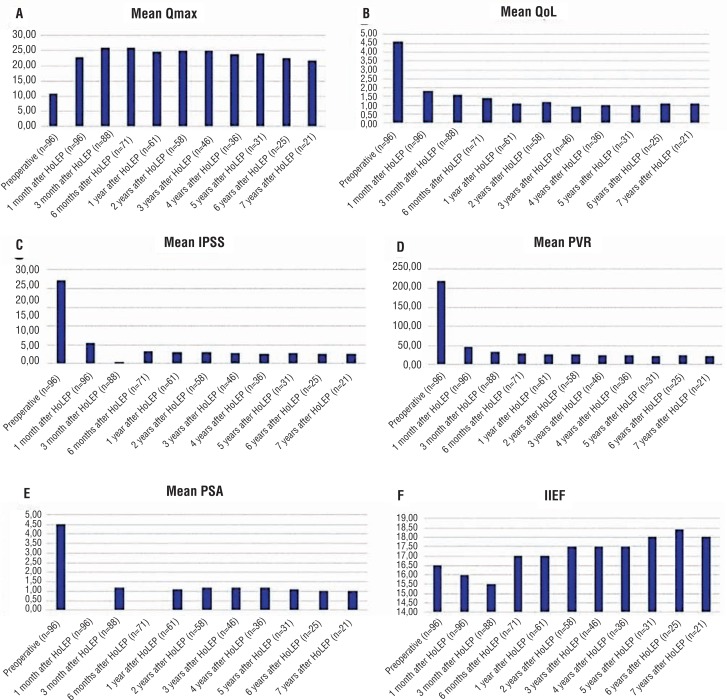
Graphics of parameters in long-term follow-up. A) Maximum flow rate in uroflowmetry, B) Quality of life, C) International Prostate symptom score, D) Post voiding residual urine volume, E) Prostate specific antigen, F) International index of erectile function, HoLEP: Holmium laser enucleation of the prostate.

Significant improvements in all Q_max_ value, QoL, PVR volume, and IPSS were evident during follow-up (all p values=0.001). Also, Q_max_ and QoL improved further every subsequent year. Dramatic decreases in PVR volume and the IPSS occurred immediately after operation. All improvements were maintained during extended follow-up. Additionally, the mean postoperative PSA level decreased significantly from the preoperative value (p=0.001). The preoperative and postoperative erectile function index scores were similar (Table-3 and Figure-2).

**Table 3:** Evaluation of parameters in follow-up period.

Parameters	Preoperative (n=96)	1 month after HoLEP (n=96)	3 months after HoLEP (88)	6 months after HoLEP (n=71)	1 year after HoLEP (n=61)	2 years after HoLEP (n=58)	3 years after HoLEP (n=46)	4 years after HoLEP (n=36)	5 years after HoLEP (n=31)	6 years after HoLEP (n=25)	7 years after HoLEP (n=21)	P value
Mean Q_max_	10.8±4.8	22.9±10.8	26.1 ±8.5	26.1 ±8.5	24.7±5.5	24.9±5.3	24.9±4.2	23.9±3.4	24.1 ±31	22.6±3.4	21.7+2.8	0.001*
Mean QoL	4.6+1.2	1.8+0.1	1.6	1.4+0.2	1.1	1.2+0.1	0.9	1+0.1	1	1.1+0.1	1.1 ±0.3	0.001*
Mean IPSS	22.1+6.1	5.5±3.8	4.3±2.9	3.3±1.7	3.1+1.7	3+1.3	2.8+1.3	2.6+1.2	2.7+1	2.5+1.1	2.6±0.9	0.001*
Mean PVR	219.8±220.9	44.6±30	33.2±18.6	27.7±15	26.1+13.4	24.8+12.7	24.6+11.1	23.6+11.3	21.4+12.1	22.4+12.1	22+13.7	0.001*
Mean PSA	4.5±0.3	N.A.	1.2+0.1	N.A.	1.1+0.1	1.2+0.1	1.2+0.1	1.2+0.1	1.1+0.1	1+0.1	1+0.1	0.001*
IIEF	16.5+6	16+5.4	15.5±5.5	17+5.4	17+5.3	17.5±5.4	175±4.9	17.5+5	18+4.3	18.5+4.4	18+4.1	0.2

**IIEF =** International index of erectile function; **IPSS =** International Prostate symptom score; **N.A =** Not assessed; **PSA =** Prostate specific antigen; **PVR =** Post voiding residual urine volume; **Q**_mal_ = Maximum flow rate in uroflowmetry; **QoL =** Quality of life; **PSA =** Prostate specific antigen.

* Statistical significant P value, Independent! tests and One way ANOVA tests were used.

The most common complication was a requirement for re-catheterisation to treat urinary retention after removing the first catheter on day 1 after surgery (seven patients). Additional non-steroidal anti-inflammatory drugs were prescribed, and catheters were re-inserted. We removed the catheters 2 days later. Eight patients (8.3%) required re-operations; three had residual adenoma, three urethral strictures, and two residual prostate tissue in the bladder (one because of morcellator malfunction and one because of a surgeon's error). All complications were of Clavien Grade 3a. Stress urinary incontinence occurred in one patient (1%); this was a Clavien 2 complication. The patient refused surgical intervention. No Clavien 3b, 4, or 5 complication developed during extended follow-up. Two patients had concomitant bladder tumours that had not invaded the muscles, and these were successfully resected during HoLEP. One tumour had just begun to infiltrate the lamina propria. These two patients were followed-up in the urology outpatient clinic. Six patients (6.25%) had bladder stones, which were laser-fragmented and removed. One patient required blood transfusion; this was a Clavien 2 complication. The two patients who were on anticoagulants were allowed to continue their medication. In two instances, the bladder mucosa was injured by the morcellation device. Coagulation of the bladder mucosa was achieved using the holmium laser.

Long-term erectile function was not affected by HoLEP; preoperative functionality neither increased nor decreased.

## DISCUSSION

Benign prostatic obstruction caused by BPH is one of the most common health problems in elderly males (12), the proportions of whom are increasing, notably in industrialised countries (13). Therefore, urologists will see more males with BPH in the future. Surgical interventions must be canvassed when medical treatments such as alpha-blockers and/or 5-alpha reductase inhibitors fail (3). Understandably, BPH patients favour minimally invasive surgical options (14). HoLEP is an acceptable option in BPH patients with indications for surgery (15). However, few long-term follow-up data are available. We focused on long–term surgical and functional results, including QoL. This is the first HoLEP study with extended follow-up from Turkey.

The present series demonstrated statistically significant improvements in Q_max_ values, QoL, and IPSS; and decreases in PSA levels and PVR volumes in extended follow-up. All of these scores are presented in Table-3. Mean Q_max_ was 22.9mL/s one month after HoLEP. Naspro et al. reported similar results; the mean Q_max_ value was 21.4mL/s after surgery (16). Yin et al. reported a significant improvement in IPSS after HoLEP (4). Krambeck et al. reported an increase in Q_max_ value (to a mean of 22.7mL/s) after HoLEP (17). QoL scores improved soon after HoLEP and continued to rise during extended follow-up. We found that QoL scores improved more than 2 times when compared to pre-operative period, one month after surgery. Sun et al. reported that QoL improved after HoLEP, being significantly better than after TURP (18). Our results are similar to those of the studies summarised above (4, 16–18). These are some of the primary end-points of our study which showed long-term effectivity of HoLEP. The fall in PSA level may be associated with prostate debulking (16).

Functional results reflected another benefit of HoLEP. Sexual functioning was neither assisted nor worsened by HoLEP. Klett et al. found that HoLEP did not adversely affect sexual function in the long-term (19). Jeong et al. reported a slight decrease in sexual functioning after operation, but this had improved 12 months later (20). However, the probability of retrograde ejaculation is 75% after operation (21). Our findings were similar with these studies (19–21). The HoLEP procedure helped to preserve sexual functions, but retrograde ejaculation still remains a problem. Improvements in voiding and sexual activity caused QoL scores to rise. Elmansy et al. reported long-term follow-up data after HoLEP (22). However, although good surgical and functional results were noted, QoL was not addressed. We have shown here that QoL improved upon long-term follow-up after HoLEP. Additionally, all improvements were maintained during long-term follow-up. Preserved functional results and improved voiding parameters are the principal findings of our present study, and constitute clinical proof of the utility of HoLEP. We strongly believe that the improvements that we recorded in various parameters reflect real clinical benefits of HoLEP.

Gupta et al. compared minimally invasive treatment options for symptomatic BPH soon after HoLEP was introduced (23). The cited authors emphasised that HoLEP afforded an ideal combination of cutting and coagulation; however, a surgical learning curve was evident (23). Thus, HoLEP needs long learning curve according to our clinical experience. Additionally, we think that at least 20 cases should be performed in view of experienced surgeon on HoLEP.

Although surgical devices and skills have improved greatly, certain complications can nonetheless develop after HoLEP. In our series, eight patients (8.3%) required re-operations; three because of residual adenomas, three to treat urethral strictures, and two to remove residual prostate tissue in the bladder. Gilling et al. found that the long-term outcomes after HoLEP were at least as good as those after TURP, which had been considered to be the gold standard endoscopic treatment option for BPH. Few complications (including re-operations) were noted in a study with 61 patients (24). Yin et al. performed a meta-analysis and noted that HoLEP afforded better results than TURP, with minimal complications (4). Our findings are similar to those summarised above. Shah et al. described the perioperative complications of HoLEP (25). Capsular perforation, superficial bladder mucosal injury, and injury to the ureteric orifice may be more prevalent when HoLEP rather than TURP is employed (25). We recorded two superficial injuries to the bladder mucosa, but no other complications. Superficial coagulation stopped the bleeding. Two patients were followed-up via routine cystoscopy, because they had bladder tumours. Only one patient required a blood transfusion. Two patients were using anticoagulants, and, because of the intrinsic nature of laser treatment, they were allowed to continue their medications. Krambeck et al. reported that HoLEP was safe for patients taking anticoagulants (5). Our findings are similar. Only one of our patients experienced stress urinary incontinence after HoLEP. Naspro et al. reported that the continence rate was high after HoLEP (16). Krambeck et al. reported less than 5% incontinence in a series of 1.000 patients (17). Our findings were similar (5, 24, 25). In view of these, HoLEP is a safe procedure with acceptable complications and presents an acceptable continence rate.

Mucosal injury can occur due to entrapment during morcellation of prostate tissue. Thus, surgeons should be aware of this. Two patients experienced mucosa injury during morcellation, in this series. Cornu et al. reported that the Lumenis^®^ device may morcellate more rapidly than the Wolf device (26). Ritter et al. reviewed the two morcellation devices in an ex-vivo model and reported limitations of these devices (27). We used both devices, and we agree with the findings summarised above (26, 27). Additionally, clinicians should be aware that morcellators can malfunction; a problem with residual prostate tissue may arise. In the present study, only a patient needed an additional operation to treat residual adenoma in the bladder. However, despite the (few) complications, HoLEP is a very acceptable minimally invasive mode of surgery when it is necessary to treat an enlarged prostate. No complication was life threatening.

The retrospective nature of our work and the low numbers of patients are limitations of the study. However, the long-term follow-up with QoL scores, and the fact that this is the first patient series from Turkey, are useful features of the present study.

Our principal goals were to document improvements in voiding parameters and QoL, and an acceptable level of manageable complications, upon extended follow-up of 96 patients who underwent HoLEP. Although the authors changed their workplaces during the follow-up time, I.A. was able to collect all long-term follow-up data because his new workplace is located in the city where the study was conducted. To the best of our knowledge, this is the first study to measure long–term QoL after HoLEP.

## CONCLUSIONS

HoLEP is safe and effective when used to treat symptomatic BPH patients. Improvements in IPSSs, Q_max_ values, and PVR volumes prove that the long-term surgical outcomes are good. Retention of sexual function, an acceptable continence rate, and improvement in QoL, are major advantages of the procedure. Thus, HoLEP can be a viable option to TURP. Multi-institutional studies are needed to gather long-term post-HoLEP data.
